# Where Does Eureka Come From? The Effect of Unreportable Hints on the Phenomenology of Insight

**DOI:** 10.3390/jintelligence10040110

**Published:** 2022-11-20

**Authors:** Artur Ammalainen, Nadezhda Moroshkina

**Affiliations:** 1 Laboratory for Cognitive Research, Russian Academy of National Economy and Public Administration, 119571 Moscow, Russia; 2 Institute for Cognitive Studies, Saint Petersburg State University, 199034 Saint Petersburg, Russia

**Keywords:** insight, creative problem solving, Aha!-experience, anagrams, semantic hints, processing fluency

## Abstract

Insight interests researchers given its special cognitive mechanisms and phenomenology (an Aha! experience or Eureka moment). There is a considerable amount of research on the effect of hints on performance in insight problem solving. However, only a few studies address the effect of hints on the subjective experiences of solvers, and the picture their results provide is unclear. We analyze the effect of unreportable true and false hints on different dimensions of the Aha! experience (subjective suddenness, Aha! experience as an effect, and certainty). Using the processing fluency framework, we predict that true hints lead to more insights and stronger Aha! experience and certainty, while false hints lead to the opposite results due to the controlled inhibition of the inappropriate representation. The results showed that false hints decreased the chance of finding a correct solution. The true-hint condition did not lead to more correct solutions but made solutions feel sudden more often than the control condition. The ratings of the Aha! experience and certainty were higher for solutions obtained after true hints than after false hints. We obtained partial support for the effect of unreportable hints on “Eureka!” moments.

## 1. Introduction

### 1.1. The Effect of Hints on Insight Problem Solving

Creative thinking requires original ideas. Such ideas might have different paths to mind, which is demonstrated by the autobiographical descriptions of scientific discoveries or inventions. It is generally accepted that people can solve problems in at least two different ways: either through step-by-step analytical processing or by sudden insight, in which a solution is reached suddenly through a reorganization of the mental representation of the problem (Sternberg and Davidson 1995). Often, insightful solutions are accompanied by an Aha! experience also called Eureka! moments (Kounios and Beeman 2014). Perhaps, the most famous model of creativity—Wallas’s four-stage model—describes insight as a key stage in the creative process (Wallas 1926; Sadler-Smith 2015). Some researchers note that in practice it can be difficult to distinguish sudden insights from discoveries in which new understanding was achieved gradually (Klein and Jarosz 2011). However, most researchers claim that insight and analytical solutions are fairly distinguishable using self-reports (Bowden et al. 2005; Danek et al. 2014; Laukkonen and Tangen 2018). Furthermore, the solvers themselves see their insightful ideas as something special. In fact, people even overrate the creativity and importance of the Aha!-like ideas (Gable et al. 2019). We can conclude that understanding creativity requires studying the nature of insights.

Autobiographical insight descriptions often include stories about accidental hints, such as the apple that fell on Newton. These stories are about external stimuli not directly related to the problems becoming the sources of insightful ideas. Seifert and colleagues suggested the concept of opportunistic assimilation to describe insight as a result of an external hint (Seifert et al. 1995). According to this hypothesis, unsuccessful attempts and an impasse are recorded in long-term memory with a special mark that the problem has not been solved. Subsequently, suppose that some external stimulus resonates with the problem marked in this way. In that case, it starts an automatic process of incorporating new information into the representation of the problem, which leads to insight.

Such theories might explain how hints determine the appearance of the ideas in the mind. Thus, the question arises: How do hints influence the chance of experiencing a Eureka! moment? In the present work, we investigate the effect of unreportable hints on *insight phenomenology*.

### 1.2. The Effect of the Hints on Cognitive Aspects of Insight Problem Solving

The restructuring representation is commonly assumed by modern theorists to be the main mechanism of insight (Ohlsson 1992; Ohlsson 2011; Danek 2018; Kounios and Beeman 2014; Weisberg 2015). Restructuring (or representational change) is an immediate change in the view of a problem as a whole (Ohlsson 1992; Ash et al. 2009). The concept was introduced to the psychology of thinking by Gestalters (Duncker 1945), who was also the first to attempt to influence restructuring using hints. The most prominent example is Maier’s seminal study (Maier 1931), in which seeing a rope swinging helped people solve a two-string problem. In this case, the swinging rope changed the representation to the “rope as a pendulum”, which eventually led to the solution. Since then, hints have become a widely used instrument to trigger the restructuring of a problem, although there are arguments against such an approach. Ash and colleagues (Ash et al. 2009) argue that the effect of hints might be explained from different perspectives, and restructuring should be investigated as a spontaneous process.

The choice of hints depends on the problems and aims of the study. In research with classical insight problems, participants are often prompted with the correct configuration of the elements or actions necessary for successful solving. For instance, Thomas and Lleras (2009) used a two-string problem, the same as in Maier’s study, and increased the solution rate by asking participants to move their arms in a manner related to the solution. Weisberg and Alba (1981) instructed participants to act outside the square in the nine-dot problem but did not find a hint effect. Chronicle and colleagues (Chronicle et al. 2001) found an effect of instruction and correct illuminating configuration in the nine-dot problem (see also the comprehensive research on the sources of difficulty in the nine-dot problem by Kershaw and Ohlsson 2004).

The effect of hints on restructuring and insight has been investigated in verbal puzzles, such as anagrams and remote compound associates (CRA) problems. In CRA problems, people are asked to produce a word that forms a compound word with three unrelated clue words (Bowden and Jung-Beeman 2003). Becker et al. (2020) provided participants with modified CRA problems in which the first words were ambiguous. Each task was preceded by either relevant or misleading priming (the word related to the correct or incorrect meaning of the ambiguous CRA word). They found that the solution rate increased while the solution time decreased after the relevant priming. Spiridonov et al. (2021) obtained the same results on similar material, prompting correct solutions by engaging participants in sentence-generation tasks. Pétervári and Danek (2019) and Danek and Flanagin (2019) increased solution rates and decreased solution times with verbal and pictorial hints in magic trick tasks.

In the mentioned studies, hints were rather overt, so participants were aware of them. However, sometimes researchers use hints that people cannot report. Bowden calls such hints unreportable, distinguishing them from reportable and undetectable. The term “reportable” refers to the hints that people are fully conscious of; hence they can report the hint’s content. “Undetectable” are those hints that cannot be identified by participants, i.e., one cannot tell whether something was presented. Unreportable hints lie somewhere in between. Even if participants notice that they were presented with some stimuli, they do not know what those stimuli were. In Bowden’s study (Bowden 1997), participants solved anagrams after being presented with unreportable hints that could be solution words, solution-related words, or unrelated words. The results indicated that presenting relevant unreportable hints had a positive effect on participants’ ability to solve anagrams. In our previous study (Ammalainen and Moroshkina 2021), we used reportable and unreportable pictorial hints for anagrams. The experiment revealed that both reportable and unreportable true hints increased solution rates and decreased solution times, while false hints increased error rates. Hattori et al. (2013) provided participants with classical insight problems, such as the nine-dot problem and the X-ray problem. Participants were interrupted after one minute and either watched a video clip containing a subliminal hint or solved simple maths problems. The solution rate was higher after they watched clips with hints.

The results described above show that most of the time hints aid problem solving by correcting the initial representation of the problem, which is assumed to be incorrect in insight problems. Hence, in most studies, the control condition is the absence of a hint or a pseudo-hint that does not relate to the problem in any way. However, the initial representation might not be incorrect (Cranford and Moss 2012), and forming inappropriate representation requires false hints, i.e., information that relates to the problem and prompts incorrect solutions. In both Becker et al. (2020) and Spiridonov et al. (2021) studies, the first words of CRA problems were ambiguous, and only one meaning of the word could be used to generate the correct solutions. The authors used hints that activated the incorrect meanings of those words and showed that the solution rate was lower while response times were longer in that case. Pétervári and Danek (2019) used false hints for magic tricks. These hints activated concepts related to the most popular incorrect solutions and, consequently, decreased solution rates and increased response times. We (Ammalainen and Moroshkina 2021) provided participants with anagrams that contained within them a word one letter shorter. False hints were pictures corresponding to those shorter words. The results showed an increase in the probability of incorrect solutions corresponding to the false hints in the false-hints condition compared to the no-hints condition. Grimmer et al. (2022), also using anagrams, showed a negative effect of primes related to incorrect solutions.

This review provides an interesting picture. True hints decrease the time needed to solve a problem correctly. However, when presented as primes before the problem, hints also decrease the chance of forming inappropriate representation and, hence, restructuring. False hints make problems more difficult, presumably because they lead to a better chance for inappropriate representation to appear. Thus, correct solutions after false hints should be obtained by restructuring more often. According to representational change theory (Ohlsson 1992), we should expect the reverse effect of hints on the phenomenology of insight. There should be fewer insightful solutions after true hints and more insightful solutions after false hints. In the next paragraph, we review the experimental data on this question.

### 1.3. The Effect of Hints on the Phenomenology of Insight

As opposed to studies considering cognitive mechanisms, few studies address the question of the effect of hints on the phenomenology of insight. Furthermore, some nuances become important when investigating this effect. The general belief is that insight cannot be achieved if a solver knows the solution was prompted. Many researchers also consider insight to be a result of unconscious processing. However, there are almost no studies in which the unreportable hints paradigm is exploited, and the Aha! experience is measured simultaneously. Bowden’s study (Bowden 1997) showed that correct solutions after relevant unreportable hints are characterized by higher suddenness ratings, which he interprets as stronger Aha! experiences. However, our previous study (Ammalainen and Moroshkina 2021) showed no increase in Aha! experience ratings for correct solutions given after true unreportable hints.

As for explicit hints, Pétervári and Danek (2019) observed an increase in feeling-of-warmth (FOW) ratings after hints were provided in magic tricks. FOW reflects the subjective suddenness of solutions. Bilalić and colleagues’ experiment (Bilalić et al. 2019) also revealed that hints to matchstick arithmetic problems led to higher suddenness and surprise rates. However, another experiment (Bilalić et al. 2021) did not show this effect for the mutilated checkerboard problem and the eight-coin problem. Davidson (1995) found a decrease in FOW ratings after cues were provided for different problems

It is often expected that correct solutions provided after false hints must be accompanied by a stronger Aha! experience since they come with the restructuring of representation. In our study (Ammalainen and Moroshkina 2021), we did not find such an effect. The results of Spiridonov et al. (2021) also showed no effect of the misleading prime on the Aha! experience. Furthermore, Becker et al. (2020) reported that solutions obtained after misleading primes were less often accompanied by the Aha! experience than solutions obtained after relevant primes.

Thus, the results of studies examining how hints influence the phenomenology of insight are heterogeneous and do not provide a clear picture. Some studies show that true hints induce more insightful solutions, while others do not. False hints increase the chance of restructuring, but their effect on the Aha! experience is either negative or absent. Importantly, these findings do not fit the predictions of the restructuring account (Ohlsson 1992; Danek 2018).

### 1.4. Why Are the Effects of Hints on Cognitive and Affective Components of Insight Inconsistent?

*1. The multidimensionality of the Aha! experience*

The experience that researchers refer to as an Aha! moment is a complex phenomenon consisting of different feelings or dimensions (Danek and Wiley 2017). Accordingly, an Aha! experience can be defined differently depending on what particular dimension is considered principal. Traditionally, most researchers consider suddenness and certainty to be definitive features of the Aha! experience. This view was implied in early studies measuring subjective insight (Danek and Flanagin 2019; Bowden et al. 2005; Jung-Beeman et al. 2004). However, there is still no agreement on which dimensions are the most significant in defining an Aha moment. Different researchers use different measures, which makes it difficult even to compare the results of the studies with each other.

Danek et al. (2014) made the first attempt to investigate the Aha! experience in detail. They provided participants with a series of magic tricks and asked them to guess the principles of those tricks. Then, participants described their Aha! experiences and rated the importance of different dimensions. The analysis showed that participants reported both cognitive (elaboration, restructuring) and emotional (happiness, tension release, drive) aspects of Aha! experience. The most important dimension of Aha! experience, according to the ratings, was happiness, while the least important was an impasse. Note that ratings were provided twice, right after the experiment and two weeks after the experiment. The ratings from the first and the second sessions were consistent with each other.

Danek and Wiley (2017) investigated which dimension, cognitive or emotional, predicts the global Aha! rating using magic tricks as problems. They showed that the strongest predictor of the global Aha! rating is a pleasure. However, other dimensions, such as suddenness, relief, and certainty also predict the global Aha! rating. Interestingly, the results of their study revealed the difference between true and false insights. While relief predicted the global Aha! rating for correct solutions, it did not for incorrect solutions. On the opposite, the surprise was only a predictor of the global Aha! rating for incorrect solutions.

Shen et al. (2016) also examined the multidimensional structure of the Aha! experience asking participants to type in emotions they had felt at the moment of insightful or analytical solutions. Then the obtained emotions associated with the Aha! experience were used in the experiment where participants chose from the list of emotions after solving a problem with or without insight. The results revealed that positive affect (feeling happy) was the most prominent characteristic of Aha! experience.

We might conclude that there are two global aspects of the Aha! experience. The metacognitive one represents the solving process and evaluation of the solution (subjective suddenness, certainty), and the effective one, which is constituted by pleasure or general positive affect. Measuring the phenomenology of insight, researchers address different aspects of it. For instance, Bowden (1997) used the suddenness scale, while Ammalainen and Moroshkina (2021) provided participants with a complex description of an Aha! experience. Both studies exploited similar stimuli (anagrams) and paradigms (unreportable hints), but only Bowden found an effect of the hints on the Aha! experience. The difference in the results of these studies might be due to their use of different measurements, which underlined different properties of the Aha! experience. Suddenness is a process feature that describes how the process of obtaining the solution was represented in the solver’s mind. The complex Aha! experience scale, although it includes the notion of suddenness, emphasizes the emotional characteristics of the result of the solving process, certainty, and positive affect.

It is also crucial to note that none of the dimensions of insight phenomenology mentioned above is specific to insight. Danek and Wiley (2020) note that suddenness, for example, might be understood by participants as a quick solution, not the solution that comes to mind all at once. Similarly, certainty judgments, although they reflect an insight phenomenology, might be additionally informed by solution time or the verification of the solution. Positive affect, being an essential part of insight phenomenology, could be biased by general positive mood. Thus, each insight measurement has a non-insight source, which means that no single measurement is a reliable way to detect insight phenomenology. The studies of the effect of the hints on insight phenomenology, therefore, have to exploit different measurements to draw more accurate conclusions.

*2. The fluency attribution hypothesis of the Aha! experience*

The Aha! experience can be described as a processing fluency effect (Topolinski and Reber 2010). According to processing fluency theory (Reber et al. 2004), subjective experiences arise due to an unexpected increase in the ease or speed of processing. Since insightful solutions are characterized by sudden comprehension, they are likely to increase the fluency of problem processing, which, in turn, is reflected in the Aha! experience. Based on this account, one might expect that hints increase the Aha! experience because they allow immediate comprehension of the solution. However, some studies do not support this hypothesis (Spiridonov et al. 2021; Ammalainen and Moroshkina 2021). One explanation for this is the fluency attribution hypothesis (Whittlesea and Williams 2001), according to which increased processing fluency must be attributed to a particular internal or external source to induce a subjective experience. According to data (Jacoby and Whitehouse 1989), knowledge about the source of changes in processing fluency neutralizes the effects of fluency. This may explain the results of Ammalainen and Moroshkina (2021). Since some hints were reportable in the study, participants were perfectly aware that they were helpful. As they knew that pictures prompted solutions, they could attribute the increased fluency to the hints even if they did not grasp the content of the pictures. A similar explanation might be applied to Spiridonov et al.’s (2021) results. The primes in Becker et al. (2020) study were only vaguely connected to the problems and left the possibility of misattribution, which explains why they found an effect of relevant primes on the Aha! experience. To avoid the attribution of fluency to external sources, we should not mix up reportable and unreportable hints when investigating the effect of the latter on the Aha! experience.

*3. Restructuring might not be sudden*

Since Gestalt psychology emerged, restructuring has been seen as a sudden event in the solving process. However, there is evidence that restructuring might take time and effort. Using verbal protocols, Fleck and Weisberg (2013) found immediate restructuring very rare. According to their data, more often, people deliberately change their representation step-by-step, gradually approaching the solution. Bilalić et al. (2019) and Ellis and Reingold (2014), using eye-tracking data, also found that solvers gained crucial information gradually. Danek et al. (2020) measured the pattern of the representational change and the Aha! experience at the same time. They found that restructuring might be gradual as well as sudden, but only the latter correlates with the Aha! experience.

Gradual restructuring might explain why solutions obtained after the provision of false hints are not accompanied by a strong Aha! experience (Becker et al. 2020; Spiridonov et al. 2021; Ammalainen and Moroshkina 2021). False hints might trigger not sudden but gradual restructuring, which involves control processes, effort, and time. In this case, we should expect a decrease in processing fluency and, consequently, a lower chance of the Aha! experience.

Thus, the effect of hints on the phenomenology of insight remains unclear. The data are controversial and, more importantly, contradict common theoretical assumptions. The processing fluency account is promising in explaining the known effects. Studies investigating this question are needed.

### 1.5. This Study

The aim of the current study is to investigate the effect of true and false unreportable hints on insight phenomenology using different measurements. Since Aha! Experience is a multidimensional phenomenon, and different judgments might be biased by information coming from the irrelevant to the insight phenomenology sources, we included two phenomenological measurements in this study. One followed the approach suggested by Bowden et al. (2005) and reflected processing features (sudden insight or step-by-step solution). Using this measurement allows us to investigate how unreportable hints affect the metacognitive awareness of the solving process. Additionally, we exploited the approach developed by Danek et al. (2014), Danek and Wiley (2017), and Shen et al. (2016), aimed to measure the emotional features of the Aha! experience (Eureka moment). This measurement refers to the affective state after the solution was obtained. The certainty about the correctness of the solution is measured separately.

We build our hypothesis using a processing fluency framework. Hence, we expected that true hints that prompt correct solutions would positively affect all subjective insight measures. False hints activate the wrong concepts in solvers’ minds. Participants are aware that this concept does not fit into the problem and exploit deliberate control processes for its inhibition. We assumed that it would lead to a decrease in processing fluency and, accordingly, negatively affect the subjective insight measures. As stimuli, we used anagrams that had one solution and contained a short word within them (e.g., anagram LACDEN, solution CANDLE, short word DANCE) and pictorial hints that referred either to a correct solution (true hints) or to a short word (false hints) (for a full description, see Ammalainen and Moroshkina 2021).

Importantly, the processing fluency caused by the hints might be attributed to the hints if participants know about them. To prevent participants from doing that, we used only unreportable hints in the experiment.

## 2. Materials and Methods

### 2.1. Participants

Justifying sample size, we were guided by our previous study with the same stimuli. A post-hoc power analysis of that experiment using the “simr” package (Green and MacLeod 2016) revealed 100% [98.17, 100] power for the effect on solution accuracy and 99% [94.55, 99.97] power for the effect on the Aha! experience. Given the effect sizes obtained in a previous study and setting alpha at .05 and beta at .9, analysis using the “sjstats” package (Lüdecke 2021) yields a required sample size of N ≥ 58. One hundred and two volunteers (79 females) with ages ranging from 18 to 57 (M = 26.04) took part in the experiment. Four participants were excluded from the analysis because they solved less than 10% of the anagrams. The final sample size was 98 participants.

All participants were native Russian speakers and had normal or corrected-to-normal vision. The experiment was conducted online. The advertisement of the experiment was distributed via VK social network (author’s personal pages and special groups for recruiting participants). The link to the experiment was accompanied by a description of the study’s aims, procedure, and the right to leave the experiment at any moment. Participants were informed that by following the link, they provided consent for participation and data usage. The experiment was conducted in accordance with the Declaration of Helsinki guidelines and approved by the Herzen State Pedagogical University Ethics Committee.

### 2.2. Materials

Twenty-four anagrams (17 had six letters and 7 had seven letters) were used for the main stage, and an additional three anagrams were used for the training. All anagrams had only one correct solution and one word within them that was shorter by one letter than the solution word. All the solution words and short words were singular Russian nouns in the nominative case. All the solution words and short words referred to a particular object or natural phenomenon. The mean frequency of solution words was 38.6 (SD = 38.0) ipm; the mean frequency of the short words was 26.2 (SD = 37.3) ipm. The hints were the images that referred to correct solutions (true hints) or short words (false hints).

### 2.3. Design and Procedure

The anagrams were presented in three different conditions: with true hints, with false hints, and without hints (in this condition, a colorful square was presented instead of a picture). Participants were randomly divided into three groups to balance the anagrams. Each participant was provided with all 24 anagrams (8 anagrams in each condition). Thus, we obtained data on all anagrams in each condition.

Before the experiment, participants read the following description of an Aha! experience (translated from Russian): “You will also need to assess how you have obtained the solution and what feelings you have experienced. One such feeling is called an Aha! experience. It is a feeling of Eureka or illumination. A prime example of the Aha! experience is the story of Archimedes, who jumped out of the bath and ran down the streets naked when he solved a problem. We do not expect you to experience such strong feelings, but if you feel something similar to an illumination (“Aha! I get it!”), consider it an Aha!-experience”.

The experiment was run online using PsychoPy 3.0.1 and Pavlovia (Peirce and MacAskill 2018). The experiment started with 3 training trials to familiarise participants with the procedure, followed by the main block of 24 anagrams. An anagram written in white text in Arial font appeared at the center of the black screen. The main block started with a fixation cross presented for 500 ms. Then, an anagram appeared for 40 s. Participants had to press the spacebar button if they solved the anagram before time ran out. A hint was presented twice: at 10 s and 25 s after the appearance of the anagram. The hints were presented for 1 frame (~17 ms). The frame rates of participants’ computers were recorded, and all were 60 Hz. The hint was followed by a mask, a circle consisting of grey pixels. If a participant pressed the spacebar, they were provided with a 7-point scale of Aha!-experience strength (−3—no Aha, +3—very strong Aha) followed by a 7-point certainty scale (−3—not certain at all, +3—very certain). Then, participants had to choose how they found the solution among the three options. We adapted instructions from Ellis and Reingold (2014) and translated them into Russian. Their English versions are as follows:

Left arrow key: “The solution came to mind suddenly, seemingly out of nowhere. I have no awareness of having done anything to try to get the answer”

(full insight)

Right arrow key: “I tried various letter arrangements to solve the anagram, but none of them seemed to work. Then, the solution came to mind suddenly”

(partial insight)

Up arrow key: “I tried various letter arrangements to solve the anagram. I was able to build on one of these arrangements to work out the solution step by step”

(non-insight)

Down arrow key: “I did not solve the anagram”.

**Figure 1:**
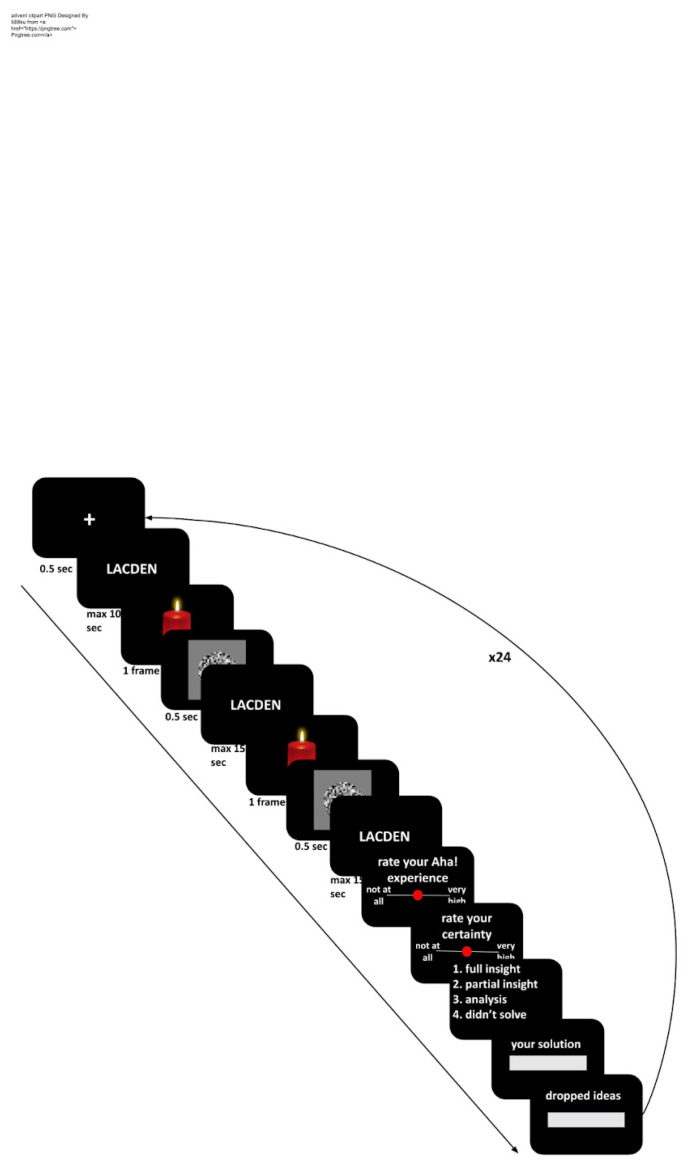
The timeline of the experimental trial (The “candle” image is taken from pngtree.com (Source 2022, accessed on 30 September 2022)).

Then, the participants could type in their solutions. There was no feedback on the correctness of the solutions. After submitting the solution, participants were asked to type in any ideas they had during the solving process but dropped. The timeline of the experimental trial is depicted in Figure 1.

For the experiment, it was not important that the hints were undetectable, but it was important that they were unreportable and participants could not relate them to the problems. Bowden (1997) showed that undetectable hints could not affect behavioral measures of anagram solving, which is consistent with contemporary research on visual perception using the Perceptual Awareness Scale (PAS) (Overgaard and Sandberg 2021). In the current study, we present hints for a single-screen refresh cycle, which was approximately 17 ms for screens with a frame rate of 60 Hz. It is consistent with Bowden’s operationalization of unreportable hints that he was presented for 16.7 ms. The frame rates of participants’ screens were recorded and were confirmed to be 60 Hz for all participants. As people have different recognition thresholds, we also used subjective reports to exclude from the analysis the trials in which participants reported they identified pictures under the masks. Twenty-one participants reported that they identified at least one picture. The trials in which people identified the hints were removed from the analysis. The number of such trials did not differ between true and false hints (18 and 13, respectively; F < 1). Although the use of subjective reports is widely criticized, it is necessary when we aim to account for participants’ subjective experiences (Overgaard and Sandberg 2021).

### 2.4. Data Analysis

The data were analyzed using RStudio (R Core Team 2017). Mixed-effect regression models were built using the lme4 package (Bates et al. 2015). Mixed-effect models were chosen because they allow addressing both between-participants and between-stimuli variance. Binary variables were modeled using a generalized linear mixed-effects model with a default logit link function. Continuous variables were modeled using linear mixed-effects models assuming a Gaussian distribution.

As the hypotheses were about the effects of the hints, solutions given before hints were excluded from the analysis (20% of all solutions). The shares of these fast solutions did not differ by hint type (F < 1).

## 3. Results

### 3.1. The Probability of Correct Solutions and Intrusion Errors

We hypothesized that true hints would lead to a higher proportion of correct solutions, while false hints would lead to a smaller proportion of correct solutions compared to the no-hints condition. Table 1 shows the shares and the numbers of correctly solved and unsolved problems across hint types before and after removing the trials with seen hints. To test the hypothesis, we built a logistic mixed-effect regression model in which the hint type, the solution frequencies, and the hint word served as fixed effects. The outcome variable was the presence of the correct solution. Participants and stimuli were modeled as random effects. The overall model was significant (χ2(2) = 5.53, *p* < .05). The model revealed a main negative effect of false hints (β = −.269, SE = .129, z = −2.079, *p* < .05). The results indicate that false hints decrease the chance of finding a correct solution. An additional model built on the data before excluding the trials with seen hints provided similar results: a negative effect of the false hints (β = −.29, SE = .13, z = −2.300, *p* < .05).

As for false hints, we expected them to lead to more specific intrusion errors, i.e., short words used as solutions. We also asked participants to type in ideas they dropped after each trial, and we expected that there would be more dropped ideas corresponding to the false hints in the false hints condition. Correct solutions were eliminated from this analysis. The data revealed an extremely low number of specific intrusion errors. Therefore, we took short words used as solutions and short words typed as dropped ideas as one unified binary variable: an intrusion. A mixed-effect logistic regression model had the hint type, the frequencies of the solution, and the word as fixed effects; the random effects were participants and stimuli. Comparison of the model with hint type as a predictor against the model without hint type showed a significant difference (χ2(2) = 16.148, *p* < .001). The model revealed a positive effect of false hints (*β* = .53, SE = .16, z = 3.217, *p* < .001), indicating that the presentation of a false hint increases the chance of a false idea appearing in the mind. Then, this idea might be used as a solution. In Table 2, the share of trials with registered intrusions across different hint types is displayed.

### 3.2. Response Times of the Correct Solutions

**Figure 2:**
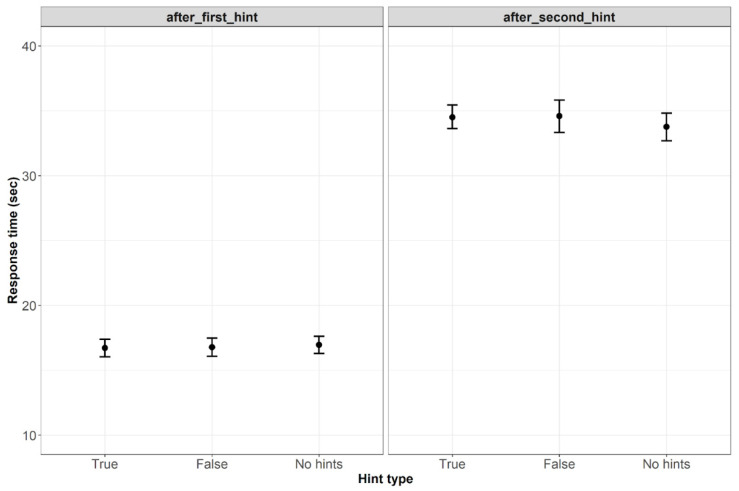
Average response times for correct solutions obtained after the first and the second hints (Bars refer to 95% confidence intervals).

We expected shorter response times for correct solutions given after true hints and longer response times for correct solutions given after false hints. As the hints were presented twice, we built two separate models for the first presentation (10–25 s interval) and the second presentation (25–40 s interval). In both models, the fixed effects were hint type, the frequencies of the solution, and short words; stimuli and participants served as random effects. The dependent variable was the response time in the 10–25 s interval for the first model and the response time in the 25–40 s interval for the second model. The model for the first time interval (10–25 s) was not significant (χ2(2) < 1) but showed an individual positive effect of the frequency of short words (β = .009, SE = .004, t = 2.01, *p* < .05), which indicates that the higher frequency of the short word is associated with longer response times in correct solutions obtained after the first hint. The model for the second time interval (25–40 s) was not significant either (χ2(2) = 2.801, *p* = .247). However, this model also revealed the positive effect of the short word frequency (β = .02, SE = .007, t = 3.09, *p* < .01). This indicates that the higher the frequency of the short word is, the more time is required to solve the anagram after the second hint was presented. Figure 2 shows the average response times for correct solutions obtained after the first and the second hints.

### 3.3. The Way the Solution Is Found

Reporting on the way they found solutions, participants in our study could choose between three options, which could be referred to as full insight, partial insight, and analysis. Since we did not investigate cognitive processes underlying insight solutions, such as representational change, it was important for us that solutions would differ in their subjective suddenness. Both full and partial insights, from our perspective, are sudden solutions. Therefore the way participants found the solution was transformed into a binary variable. Two categories of responses (full and partial insights) were merged into one category, insight, with analytical solutions comprising the opposite category.

We presumed that correct solutions would be judged as insightful more often after true hints were provided than when no hints were provided. To control for the possible time effects on insight judgments, we included the response time as a predictor. The binary variable (insight/analysis) was the dependent variable in the mixed-effect logistic regression model. The fixed effects were hint type and response time. Participants and stimuli were modeled as random effects.

The model with hint type as a predictor significantly differed from the model without hint type (χ2(2) = 12.011, *p* < .01). The model showed a positive effect of the true hint (β = .62, SE = .23, OR = 1.87[1.18, 2.95], z = 2.667, *p* < .01) and a negative effect of response time (β = −.02, SE = .01, OR = .98[.97, .99], z = −2.011, *p* < .05). The results indicate that correct solutions obtained after true hints have a higher chance of being judged as insights than solutions obtained without hints. Additionally, the faster the solutions are, the more chance they can be judged as insights.

**Figure 3:**
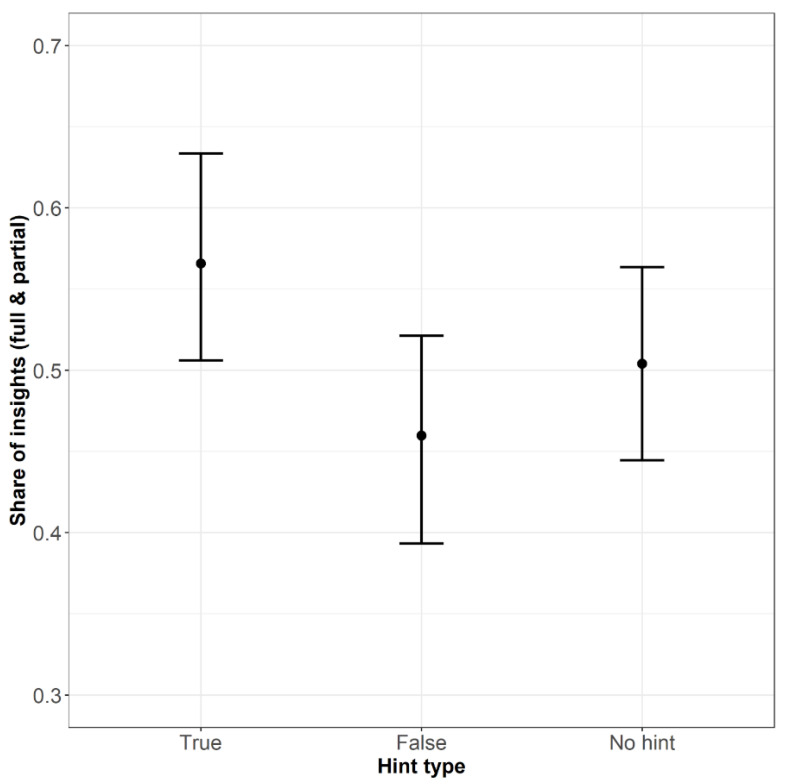
The Share of insightful solutions across conditions (Bars refer to 95% confidence intervals).

We built another model with the true hint as an intercept to see how insight judgments after false hints differ from those after true hints. The model differed significantly from the null model (χ2(2) = 12.011, *p* < .01). It revealed negative effects of both the false-hint (β = −.81, SE = .25, OR = .44[.27, .72], z = −3.260, *p* < .01) and no-hint (β = −.62, SE = .23, OR = .54[.34, .85], z = −2.667, *p* < .01) conditions as well as a negative effect of response time (β = −.02, SE = .01, OR = .98[.96, .99], z = −2.011, *p* < .05). The second model showed that the probability of insightful solutions is higher for true hints than for both false hints and no hints. The shares of insightful solutions for different types of hints are shown in Figure 3.

### 3.4. Aha!-Experience Ratings

We expected that true hints would increase and false hints would decrease Aha!-experience ratings. A mixed-effect linear regression model with Aha!-experience ratings as a dependent variable was built. The fixed effects were hint type, response time, solution frequencies, and short words. Participants and stimuli served as random effects. The no-hint condition was modeled as an intercept.

**Figure 4:**
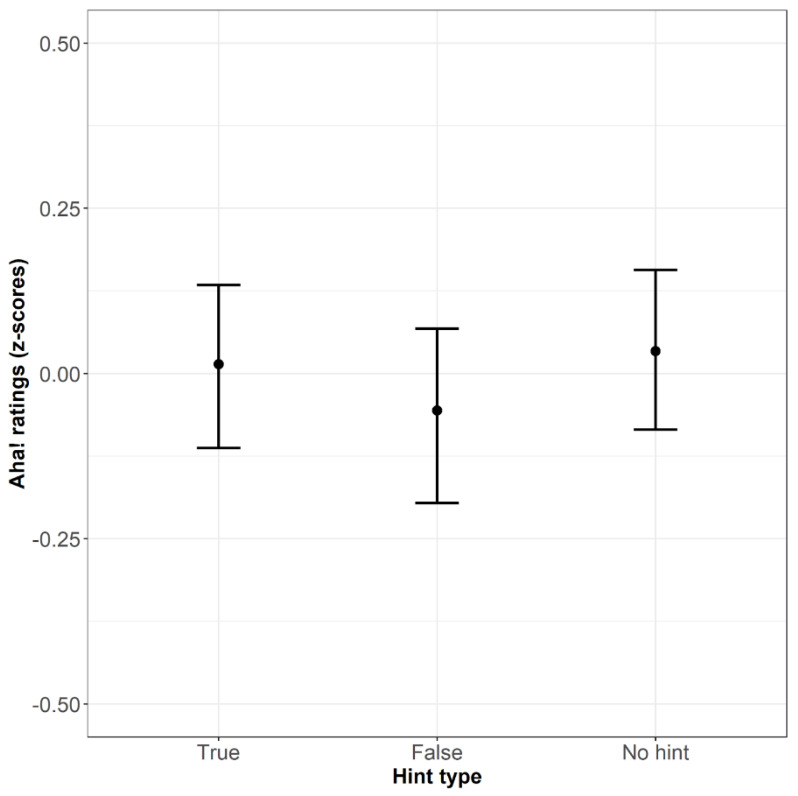
The mean Aha!-experience ratings (z-scores) across conditions (Bars refer to 95% confidence intervals).

The model with hint type as a predictor did not differ significantly from the model without this predictor (χ2(2) = 4.605, *p* = .10). We built another model with the true hint as an intercept. The model was also not significant (χ2(2) = 4.605, *p* = .10) but showed an individual effect of the false hints (β = −.26, SE = .01, t = −2.152, *p* < .05). This indicates that Aha!-experience ratings are lower for solutions given after false hints than after true hints. Figure 4 shows the average Aha!-experience ratings across different hint types.

### 3.5. Certainty Ratings

Our expectations for the certainty ratings were the same as those for the Aha!-experience ratings, so the model was identical except for the outcome variable. The model with the no-hint condition as an intercept was significant (χ2(2) = 7.816, *p* < .05) and revealed a negative effect of response time (β = −.03, SE = .004, t = −6.625, *p* < .001). This result indicates that certainty ratings were higher for faster solutions.

**Figure 5:**
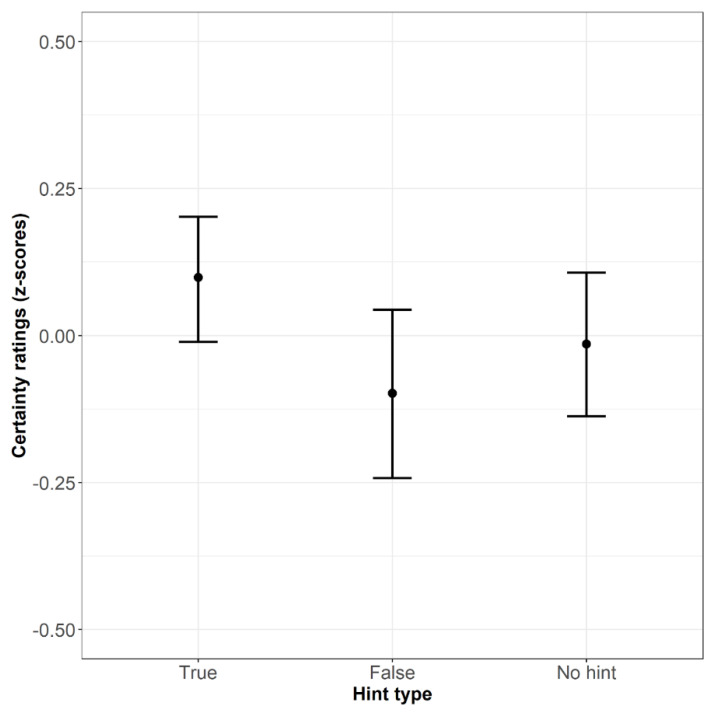
The mean certainty ratings (z-scores) across conditions (Bars refer to 95% confidence intervals).

The model with the true-hint condition as an intercept differed from the null model significantly (χ2(2) = 7.816, *p* < .05) and showed an additional negative effect of the false hint (β = −.22, SE = .08, t = −2.796, *p* < .01), which means that correct solutions receive lower certainty ratings after false hints than after true hints. The average certainty ratings are displayed in Figure 5.

## 4. Discussion

The main goal of the experiment was to investigate the effect of unreportable semantic hints on the cognitive and affective components of insight. First, we expected semantic hints to influence the behavioral measures of anagram solving. True hints would lead to more correct solutions and faster response times, while false hints would lead to fewer correct solutions, longer response times, and more incorrect ideas corresponding to false hints. Second, we proposed phenomenological hypotheses. We assumed that true hints would lead to more insightful solutions and higher Aha! experience and certainty ratings. Our expectations about false hints were the opposite.

There was partial evidence for the behavioral hypotheses. Unlike previous studies (Bowden 1997; Ammalainen and Moroshkina 2021; Hattori et al. 2013), our results did not show a positive effect of true unreportable hints on solution rate or response time. However, we found that false hints increase the chance for false ideas to appear and decrease the probability of the problem being correctly solved. Thus, unreportable hints affected the solving process, but their effect was detected only for the false hints. Explaining this observation demands further research. The results support our hypothesis that the false hints influenced solvers’ representations of the problems. Perhaps, the alternative interpretation of the anagrams (the short words within the anagrams) induced the Einstellung effect, as in Ellis and Reingold (2014). Additionally, we found that the higher frequency of the short word, the more it hinders the search for the correct solution increasing the search time. It is in line with those of previous studies that show that misleading semantic hints or priming lead to fewer correct solutions (Becker et al. 2020; Spiridonov et al. 2021) and might induce false solutions (Ammalainen and Moroshkina 2021; Grimmer et al. 2022). However, it is worth noting that only reportable hints were used in previous studies, while we found a similar effect using unreportable false hints. Notably, in this study, the share of intrusion errors, i.e., false ideas used as solutions, was low, which means that participants probably managed to check their ideas before submitting solutions. This explanation is also supported by the high certainty ratings (2.2–2.4 on average).

In our previous study (Ammalainen and Moroshkina 2021), we used reportable hints and unreportable hints and did not find an effect of the hints on Aha!-experience ratings. We assumed that the multidimensionality of the Aha! experience might be the reason why the results of our study differed from those obtained by Bowden (1997). In the present study, we used three separate scales referring to insightfulness as subjective suddenness, Aha! experience as affect and certainty. The results showed that true hints increased the chance for correct solutions to be judged as subjectively sudden (insights). Thus, even though data do not reveal the expected effect of the true hints on behavioral measures, we found the effect of the hints on the subjective suddenness judgments. This result is in line with Bowden (1997), as insights in our study were defined as solutions suddenly popping into the mind. Insight judgments were additionally associated with faster solutions. This corresponds to the idea that people can perceive suddenness as the time needed for a solution to appear and not as a solution coming all at once, as noted by Danek and Wiley (2020). Notably, our study revealed that unreportable true hints affect subjective suddenness judgments regardless of response times. It might be interpreted that the speed of solutions and unreportable hints have an additive effect on insight experience. The share of insightful solutions was higher after true hints than after false hints as well, which might be interpreted as evidence for gradual restructuring after false hints. Many authors use false hints to induce the restructuring of problems (Becker et al. 2020; Spiridonov et al. 2021; Pétervári and Danek 2019; Ammalainen and Moroshkina 2021) and expect it to be sudden. However, our data suggest that restructuring induced by false hints might actually be gradual and deliberate.

The results did not show an increase in Aha! ratings in the true-hints condition compared to the control condition. This supports the idea that different measurements of insight phenomenology have different sensitivity to the effect of the hints. The suddenness scale (similar to the one from Bowden (1997)) provided results corresponding to Bowden’s study. The Aha! experience scale, as in Ammalainen and Moroshkina (2021), provided the same results as our previous study. It could be that processing fluency caused by the hints is only reflected in suddenness judgments, i.e., the representation of the solving process. Another explanation would be that the suddenness measure (insight vs. step-by-step solution) is easier for participants to understand and, therefore, more sensitive than the Aha! experience scale, at least in anagram solving. One can assume that subjective suddenness and Aha! experience as an effect is not the same thing. The data on the relationship between these subjective judgments and solution time are consistent with this notion. We showed that faster solutions are more likely to be subjectively sudden, which is in line with previous data (Cranford and Moss 2012). However, not all studies demonstrate faster response times for insight solutions (Jung-Beeman et al. 2004). Some researchers obtain the opposite correlation when stressing the affective features of the Aha! experience in the description provided to the participants (Moroshkina et al. 2022; Stuyck et al. 2021). Accordingly, we did not find a correlation between response time and Aha! experience ratings. Perhaps, unreportable hints could influence subjective suddenness but not the emotion of “Eureka!”

Both Aha! experience and certainty ratings were higher in the true-hints condition than in the false-hints condition (this result is in line with Becker et al. (2020)). The low Aha! experience ratings might be evidence that false hints induce gradual and deliberate restructuring that does not lead to an increase in processing fluency. However, since neither of the conditions differed from the control condition, our data do not allow us to say whether Aha! experience and certainty ratings were increased by true hints or decreased by false hints or whether both effects were present. Certainty ratings were also associated with smaller response times. This result is consistent with the relationship between response time and insight judgments because insight solutions are often characterized by high certainty about their correctness (Danek and Wiley 2017; Topolinski and Reber 2010).

Certain limitations of our study have to be mentioned. First of all, the experiment was conducted online, which means we did not control for the experimental environment, such as the place, the distance between a participant and the screen, etc. It could affect the results, although within-subject design should have flattered these issues to a certain extent. Secondly, we did not differentiate between full and partial insights. However, it might be important to look closely at the difference between “full insights” and insightful solutions that were preceded by several analytical steps. Another limitation is that we used anagrams, which are convenient to experimentally study insight but are not alike complex creative problems from the real world. This imposes constraints on, for example, the practical implementation of the results. Further research is needed to expand our conclusions on more realistic problem situations.

## 5. Conclusions

We obtained partial evidence for our hypotheses. We showed that false hints influence the representation of an anagram, inducing incorrect ideas and decreasing the chance of finding a correct solution. Correct solutions given after true hints differ subjectively from correct solutions obtained without hints. They are more likely to be felt as sudden insights. However, such solutions did not differ from those obtained without hints in Aha! experience and certainty ratings. It stresses the importance of using different measures of Aha! experience since it has different dimensions. True hints led to higher Aha! experience and certainty ratings than false hints. This might be due to the increased processing fluency after true hints, the deliberate restructuring after false hints, or both of these factors. In conclusion, we may say that external unreportable hints might contribute to the feeling of a sudden discovery. However, we may also ask which measurement of insight phenomenology is the essential one. Further research should test and validate the sensitivity and relevance of different insight measurements.

## Figures and Tables

**Table 1:** Share of correct solutions across different hint types.

	Including Trials with Seen Hints		Excluding Trials with Seen Hints	
Hint type	Correctly solved	Not solved (omissions and intrusions)	Correctly solved	Not solved (omissions and intrusions) ^1^
True	.45	.55	.42	.58
False	.37	.63	.37	.63
No hint	.43	.57	.43	.57

^1^ Omissions are trials where no solution was submitted, and intrusions are incorrect solutions.

**Table 2:** Share of trials in which intrusions (either incorrect solutions or dropped ideas) were registered across different hint types.

Hint Type	Intrusion	No Intrusion
True	.13	.87
False	.21	.79
No hint	.15	.85

## Data Availability

The data generated and analyzed in this study are available at https://osf.io/mhb56/ (for access, please email the corresponding author).
